# Some light shed on *Galium transcarpaticum* (Rubiaceae) from the Ukrainian Carpathians

**DOI:** 10.3897/phytokeys.278.201206

**Published:** 2026-08-05

**Authors:** Andriy Novikov, Viktor Nachychko, Oleksandr Zinenko

**Affiliations:** 1 State Museum of Natural History of the NAS of Ukraine, Lviv, Ukraine Ivan Franko National University of Lviv Lviv Ukraine; 2 Ivan Franko National University of Lviv, Lviv, Ukraine V.N. Karazin Kharkiv National University Kharkiv Ukraine; 3 V.N. Karazin Kharkiv National University, Kharkiv, Ukraine State Museum of Natural History of the NAS of Ukraine Lviv Ukraine

**Keywords:** *
Galium
*, herbarium DNA, Rubiaceae, taxonomy, Ukrainian Carpathians

## Abstract

*Galium transcarpaticum* was described in 1979 as an endemic occurring exclusively in the Ukrainian Carpathians. Since then, little has been contributed to its taxonomy and distribution, and only a few specimens have been collected. We successfully isolated DNA and amplified nuclear (ITS) and plastid (*trn*H-*psb*A, *atp*B-*rbc*L, *trn*L (c-d), and *trn*L (e-f)) markers from *G. transcarpaticum* isotype specimens deposited at the LWS herbarium. The obtained results do not allow delimiting *G. transcarpaticum* as an independent species, but rather place it within *G. mollugo*. Hence, the new combination Galium mollugo subsp. transcarpaticum (Stojko & Tasenk.) A.V.Novikov, Nachychko & Zinenko is proposed.

## Introduction

The genus *Galium* L. s.l. has traditionally been regarded as one of the largest genera of the family Rubiaceae Juss., comprising ca. 650 species (POWO 2026), many of which are polymorphic and widely distributed (Soza and Olmstead 2010). It is classified within subfamily Rubioideae Juss., tribe Rubieae Baill. Molecular phylogenetic studies demonstrated that *Galium* s.l. was not monophyletic. In particular, Soza and Olmstead (2010) recovered seven major clades within Rubieae, with *Galium* species occurring in three of them. Subsequent analyses confirmed the non-monophyly of *Galium* s.l. (Ehrendorfer and Barfuss 2014; Ehrendorfer et al. 2018; Yang et al. 2018, 2022). Hence, recently, Kadereit and Schneeweiss (2026) proposed a revised classification of the tribe Rubieae, segregating several lineages formerly included in *Galium* s.l. and recognizing *Galium* s.str. as a monophyletic group consisting of ca. 300 species. Although *G. mollugo* L. was included in the broad-scale phylogenetic analysis of Soza and Olmstead (2010), *G. transcarpaticum* Stojko & Tasenk. has not been included in any of previous molecular phylogenetic studies.

*Galium transcarpaticum* is a problematic species, a representative of polymorphic *G. mollugo* s.l. delimited based on a complex of variable morphological features, i.e., lack of pubescence, oblanceolate leaves with acute apex and indentate margin, and the brown to blackish color of the stamens (Stojko and Tasenkevich 1979). Originally, it was described by Ukrainian botanists Stepan Stojko (1920–2020) and Lydia Tasenkevich (b. 1948) as an Eastern Carpathian endemic. However, since the species description, no occurrences have been reported outside the Ukrainian Carpathians. Consequently, Kliment et al. (2016) designated it as an Eastern Carpathian endemic occurring exclusively in the Ukrainian Carpathians.

In the original description, Stojko and Tasenkevich (1979) noted that *Galium transcarpaticum* belongs to the *G. mollugo* complex and is morphologically closest to *G. suberectum* Klokov. The latter was later nested within *G. album* Mill. as a subspecies (i.e., G. album subsp. suberectum (Klokov) Michalk.) and reported as also having glabrous and oblanceolate leaves (Michalková 1993a, 1993b). Gynda (2004) noted that *G. transcarpaticum* differs from G. album subsp. suberectum based on the flower, pedicel, and leaf size. However, no clear distinguishing size limits were provided. Besides this, Gynda (1993, 2004) indicated *G. transcarpaticum* as a diploid (2*n* = 2x = 22) and *G. suberectum* as a tetraploid (2*n* = 4*x* = 44).

Although *Galium transcarpaticum* is often cited as an endemic species represented in the flora of the Ukrainian Carpathians (e.g., Kobiv 2010; Chorney 2011; Tasenkevich et al. 2025), only a few specimens were collected since its description and deposited at the herbaria KW (holotype), LWS (isotypes), KWHA, and CHER (see Thiers 2026 for herbarium acronyms). In the “Flora of the Ukrainian Carpathians” (Chopyk and Fedoronchuk 2015), *G. transcarpaticum* is not listed at all. Instead, it is mentioned in a recent checklist by Fedoronchuk and Shyian (2025), with reference to the old herbarium material and the initial publication by Stojko and Tasenkevich (1979).

The lack of clear delimiting characteristics of *Galium transcarpaticum* led to uncertainty about its taxonomic status and raised questions about whether it represents an independent species or an intraspecific morph within the *G. mollugo* complex.

At the herbarium LWS, we were able to sample herbarium material of *Galium transcarpaticum* and *G. mollugo*, collected by Lydia Tasenkevich herself. Therefore, these specimens represent authentic material, allowing for the distinction of *G. transcarpaticum* from *G. mollugo* by Lydia Tasenkevich, one of the authors of the former species. Unfortunately, we were unable to obtain authentic material of G. album subsp. suberectum as the rules of the KW herbarium, where it is stored, do not allow such destructive sampling. At the same time, the LWS herbarium does not contain such authentic specimens. Here, we present our brief outcomes and considerations based on the combined analysis of nuclear and several plastid markers.

## Material and methods

For molecular analysis, the material (5–10 leaves) has been sampled from six herbarium specimens of *Galium transcarpaticum* (three isotypes, two specimens from the locus classicus, and one specimen from the surrounding mountain area) and three specimens of *G. mollugo*, deposited at the LWS herbarium and collected in the Ukrainian Carpathians (Suppl. material 1). The material was homogenized in a ceramic mortar with an excessive amount of lysis buffer. The total DNA has been isolated from the herbarium material using the EURx GeneMATRIX Plant & Fungi DNA Purification Kit. Then the nuclear (ITS), and plastid (*trn*H-*psb*A, *atp*B-*rbc*L, *trn*L (c-d), *trn*L (e-f), *trn*C-*ycf*6, and *rpo*B-*trn*C) regions were amplified using the Thermo Scientific DreamTaq PCR Master Mix (2X), and applying the primers and programs listed in Suppl. material 2. Amplification success was analyzed using the electrophoresis in the agarose gel. As a result, five DNA regions, (i.e., ITS, *trn*H-*psb*A, *atp*B-*rbc*L, *trn*L (c-d), and *trn*L (e-f)) were successfully amplified for three *G. transcarpaticum* and three *G. mollugo* specimens (Suppl. material 1). For successfully amplified samples, the standard Sanger sequencing was performed by an outsourcing company (Macrogen). Additional sequences for *G. aparine* L. (served as an outgroup), as well as for *G. mollugo* and *G. album* (served as comparative reference sequences), were downloaded from GenBank (see Suppl. material 1 for the records details). The obtained sequences were aligned and concatenated using Geneious Prime.

The Bayesian Inference analysis was performed using MrBayes 3.2.6 in the PhyloSuite 2 environment (Zhao et al. 2025). Two matrices were applied independently: (a) with nucleotide sequences only and (b) a partitioned dataset consisting of nucleotide characters (positions 1–1801) and 0/1 coded indels (positions 1802–1807). For the gap analysis, the concatenated sequences were initially processed with FastGap 1.2 (Borchsenius 2009). After that, only informative gaps were selected and built into the partitioned dataset. For the nucleotide partition, the GTR+Γ substitution model was applied. The indel partition was analyzed as standard discrete morphological characters using variable coding and gamma-distributed rate variation. Model parameters were unlinked across partitions. Two million generations of Markov chain Monte Carlo were run with four chains and a temperature parameter of 0.2, sampling trees every 1000 generations. The first 500 sampled trees were discarded as burn-in prior to constructing the majority-rule consensus tree and estimating posterior probabilities. The obtained trees were visualized using FigTree v1.4.4 software (Rambaut 2018).

Morphological analysis has been performed on the specimens deposited in the LWS herbarium using a standard ruler, caliper, and stereomicroscope Kern OZM-983.

The distribution map was visualized using QGIS 3.10.2 and was based on consolidated data from the LWS, KWHA, and CHER herbaria (Suppl. material 4). The specimens of *G. transcarpaticum* deposited at the LWS herbarium were digitized and can be accessed online (herbUA 2026). The specimens examined, in general, lack collectors’ personal numbers. Moreover, in some cases, herbarium accession numbers were not assigned, as in the KW, KWHA, and CHER herbaria. Accordingly, such specimens are listed here as unnumbered (s.n.).

## Results and discussion

The DNA extracted from the herbarium material, especially that exposed to regular high-temperature treatments for years, as in the LWS herbarium, is often degraded (Staats et al. 2011; Porrelli et al. 2026). This generally raises concerns about working with such material, increases the workload, and limits the application of standard molecular markers. Therefore, in our research, we focused on the application of markers resulting in sequences up to 1 kbp in length. We were unsuccessful in amplification of *trn*C-*ycf*6 and *rpo*B-*trn*C plastid regions. However, other applied markers worked well with most of the treated specimens.

**Figure 1.**
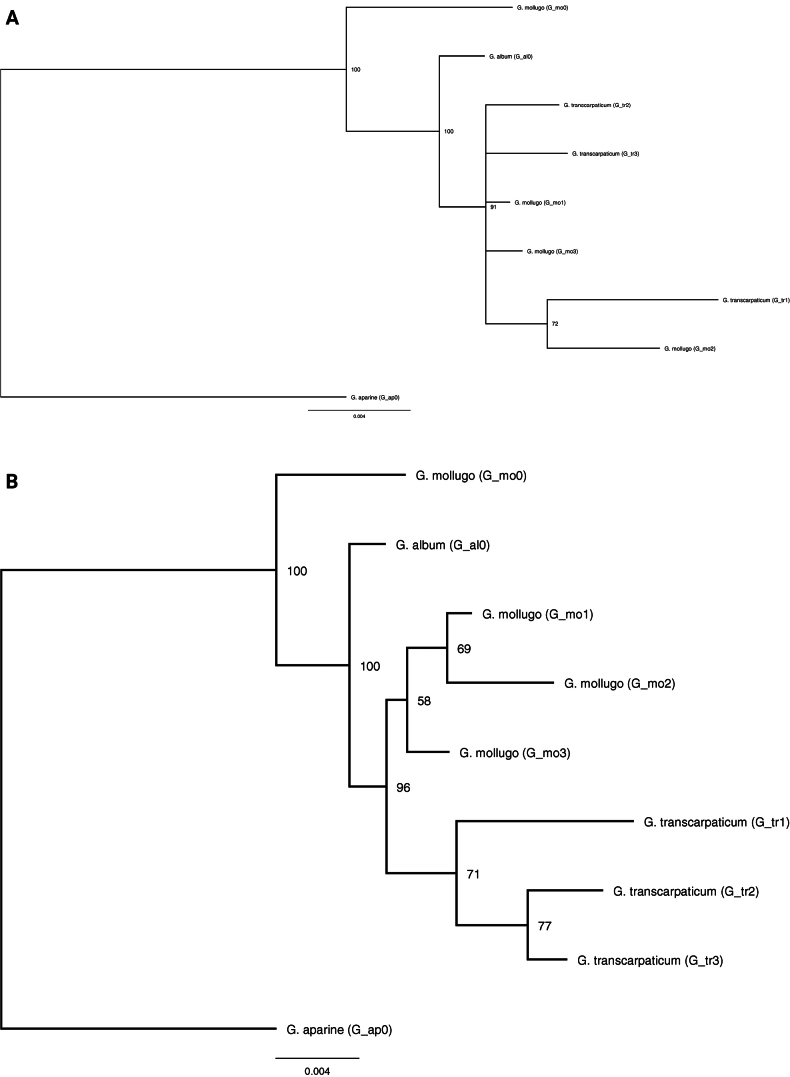
The results of Bayesian analysis of *Galium transcarpaticum*: **A**. nucleotide data only; **B**. nucleotide data combined with coded indels.

The received fragment of the *ITS* sequence (223 bp) failed to distinguish between Ukrainian *Galium transcarpaticum* and *G. mollugo*. Similarly, a 304 bp sequenced fragment of the *trn*H-*psb*A spacer did not show any differences between Ukrainian *G. mollugo* and *G. transcarpaticum* samples. The *trn*L-F region amplified with c-d (520 bp) and e-f (389 bp) primers did not show meaningful differences between *G. transcarpaticum* and *G. album*, or between *G. transcarpaticum* and *G. mollugo*, consistently placing them in the same clade with very little internal structure. The only observed differences of *G. transcarpaticum* from *G. mollugo* have been observed in the 634 bp sequence of *atp*B-*rbc*L chloroplast spacer, containing one unique insertion (in the position 54829 of *G. mollugo* plastid complete genome NC_036970.1) and two deletions in the positions 54971 and 55075. While indels are not considered informative characters by most phylogenetic algorithms, they represent here three independent evolutionary events and an apomorphy of the *G. transcarpaticum* lineage. Therefore, in addition to the traditional analysis of nucleotide sequences (Fig. 1A), we performed combined Bayesian analysis of nucleotide sequences and indels (Fig. 1B).

In the Bayesian analysis based solely on nucleotide substitutions, *Galium transcarpaticum* was not recovered as a monophyletic group, as its accessions were intermixed with representatives of *G. mollugo* specimens from the Ukrainian Carpathians. This result indicates very close molecular affinity between the two taxa and does not support clear species-level separation. After the inclusion of coded indel characters, the tree topology slightly changed, but the overall result remained the same. All sampled accessions of *G. transcarpaticum* formed a clade distinct from the clade of Ukrainian *G. mollugo*. However, support for the *G. transcarpaticum* clade was moderate (PP = 0.71), while a strong support value (PP = 0.96) was gained for the clade containing both *G. mollugo* and *G. transcarpaticum*. Thus, although coded indels provide an additional phylogenetic signal and suggest some molecular differentiation of *G. transcarpaticum*, this evidence remains limited and does not allow to confirm *G. transcarpaticum* as an independent species.

In both phylogenetic trees obtained (Fig. 1A, B), the sequence assigned to *Galium album* was placed within the broader lineage containing *G. mollugo* and *G. transcarpaticum*. *Galium album*, including *G. suberectum* (currently treated as G. album subsp. suberectum) is tetraploid, whereas *G. mollugo* (like *G. transcarpaticum*) is diploid (Michalková 1993a, 1993b; Gynda 1993, 2004). Nevertheless, these taxa may sometimes be confused morphologically. The observed placement of *G. album* may therefore reflect specimen misidentification, limited phylogenetic resolution of the analyzed markers, or a close evolutionary relationship among these taxa. Although resolving this issue requires further investigation, it lies beyond the scope of the present study. Nevertheless, this result raises concerns about the reliability of molecular data deposited in public databases and highlights the importance of using traceable sequences linked to verifiable herbarium vouchers. For this reason, our study included material collected by Lydia Tasenkevich to assess the relationships among specimens that she identified as *G. transcarpaticum* and clearly distinguished from *G. mollugo*. The GenBank sequences labelled G_mo0, G_al0, and G_ap0 were included only to provide broader phylogenetic context and were not used for species delimitation or for interpreting relationships among our focal specimens.

Morphological analysis showed that *G. transcarpaticum* shares most of its characters with *G. mollugo* (Suppl. material 3), which is consistent with the close relationship revealed by the molecular data. Nevertheless, differences were observed in several morphological characters, although their ranges partly overlap or form a continuum with those in *G. mollugo* (Table 1). In particular, *G. transcarpaticum* plants are generally smaller and have shorter and narrower basal leaves, a greater number of leaves per node, and larger flowers and fruits. Mature fruits of *G. transcarpaticum* are normally dark brown, whereas those of *G. mollugo* are generally lighter and reddish-brown. None of these characters alone provides an absolute distinction between these taxa. However, the combination of indicated characters allows to distinguish *G. transcarpaticum* from *G. mollugo*. The morphological differentiation of *G. transcarpaticum* is also accompanied by its distinct ecological pattern. *Galium transcarpaticum* is associated with open limestone outcrops in the montane belt, where it occupies relatively dry, nutrient-poor, isolated and low-competition habitats. By contrast, *G. mollugo* has a much broader ecological amplitude and is commonly found in more mesic and nutrient-rich habitats, including meadows, forest glades, and forest margins. The geographically restricted distribution and specialized habitat preferences of *G. transcarpaticum* may therefore have contributed to the maintenance of its morphological distinctiveness despite its close genetic affinity with *G. mollugo*.

**Table 1.** Comparative assessment of *Galium transcarpaticum* and *G. mollugo* in the Ukrainian Carpathians.

**Characters**	***G. transcarpaticum***		***G. mollugo***	
	**Protologue (Stojko and Tasenkevich 1979)**	**Analyzed specimens**	**Description of Michalková (1993b)**	**Analyzed specimens**
Main shoot length	(15–) 30–40 (–45) cm	25–34 cm	(40–) 60–70 (–140)	60–140 cm
Number of leaves per whorl	6– (7–9) –10	6–8	5–7 (based on illustration)	5–6
Basal leaves length	0.5–1.0 cm	1.0–1.2 cm	–	1.2–3.0 cm
Basal leaves width	–	1.1–1.5 mm	–	1.6–3.4 mm
Perianth diameter	3.5–5.5 mm	2.5–3.7 mm	(2.3–) 2.4–2.7 (–3.5) mm	1.7–2.5 mm
Fruit length	Up to 2 mm	1.0–1.8 mm	(0.9–) 1.0–1.2 mm	0.9–1.2 mm
Fruit color	Dark brown	Dark brown	Reddish-brown	Reddish-brown
Substrate	Lime outcrops	Lime outcrops	Eutrophic moister alluvial soils	Eutrophic moister soils

Limestone outcrops form fragmented and isolated landscapes that support high levels of species and genetic diversity (Taberlet et al. 2012). Davis (1951) noted that cliffs and rocky habitats often serve as refugia and are therefore rich in chamaephytic relicts, many of which can persist in geographic isolation. At the same time, Csergő et al. (2014) argued that geographic distance is an important driver of genetic and species turnover in such habitats. Knotek and Kolář (2018) demonstrated that low-competition habitats of various substrates and elevations may play a principal role in preserving genetic diversity of isolated populations of the *G. pusillum* complex in mountains of Central Europe. They noted that the ecologically stable habitats of the interglacial refugia may buffer the genetic drift and, consequently, prevent significant diversity loss. Hence, it is reasonable to suggest that *G. transcarpaticum* may occur outside the Ukrainian Carpathians within other similarly isolated habitats. In a personal communication, Clemens Pachschwöll of the University of Vienna suggested that *G. transcarpaticum* may occur in the Romanian Carpathians. Given the numerous limestone outcrops in this region, such a scenario appears plausible. However, no occurrences of *G. transcarpaticum* have yet been reported from the Romanian Carpathians.

**Figure 2.**
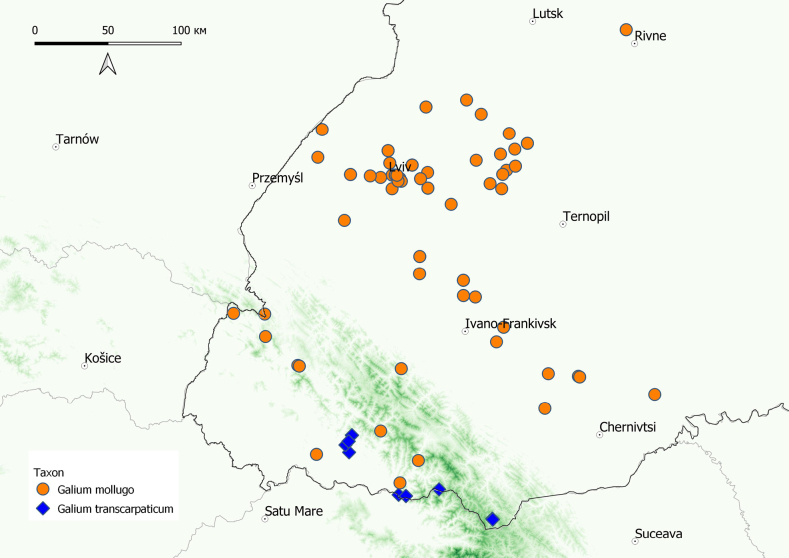
Distribution of *Galium transcarpaticum* and *G. mollugo* in the Ukrainian Carpathians and adjacent lowlands based on herbarium data. The map is based on the list of examined specimens given in Suppl. material 4.

Considering the general results of the molecular analysis presented here, as well as the shared distribution range (Fig. 2), diploid chromosome number, and overall morphological similarity, we conclude that *Galium transcarpaticum* should be treated within *G. mollugo*. At the same time, the presence of diagnostic indels, distinct ecological preferences (particularly its association with limestone outcrops), and the morphological differences summarized in Table 1 support its recognition at the subspecies level. Therefore, we propose the following new combination: G. mollugo subsp. transcarpaticum (Stojko & Tasenk.) A.V.Novikov, Nachychko & Zinenko.

### Galium mollugo subsp. transcarpaticum

Taxon classificationPlantaeGentianalesRubiaceae

(Stojko & Tasenk.) A.V.Novikov, Nachychko & Zinenko comb. et stat. nov.

E3D72ADF-446F-5897-B1CD-7254F16D16DE

urn:lsid:ipni.org:names:77393670-1

Galium transcarpaticum Stojko & Tasenk., Ukrayins’k. Bot. Zhurn. [Ukr. Bot. J.] 36(6): 594. 1979. [Basionym]

#### Type material.

RSS Ucrainiae, Carpati Orientales Ucrainici, Jugum Poloninense, districtus Transcarpatia, pagus Velika Ugolyka, rupes calcarea in loco Velikij Grebenj, 820 m, in clivis austrooccidentalibus. Leg. Stojko et Tasenkevitsch, 27.07.1976 (Stojko and Tasenkevich 1979). ***Holotype***: KW (s.n.). ***Isotypes***: LWS 017273, LWS 112550, LWS 112551.

#### Distribution and habitat.

Ukrainian Carpathians. Open lime outcrops in the upper mountainous vegetation belt at the elevations of 800–1100 m a.s.l. Xerothermic and mesophytic calcareous communities.

#### Selected specimens examined.

RSS Ucrainiae, Carpati Orientales Ucrainici, Jugum Poloninense, distr. Transcarpatia, pagus Velika Ugoljka, rupes calcarea, in loco Velikiy Grebinj, 820 m, in clivis austro-occidentalibus: 1976-07-27 Stoyko S.M. & Tasenkevich L.O. (Holotype – KW s.n.; Isotypes – LWS 112550, LWS 112551) • Zakarpattia region, Tiachiv district, Uholka forestry of Carpathian State Reserve, lime slope of Hrebin, 820 m a.s.l., SW slope: 1976-07-27 Stoyko S.M. & Tasenkevich L.O. (Isotype LWS 017273) • Zakarpattia region, Tiachiv district, Uholka massif of the Carpathian Nature Reserve. Lime rocks: 1988-06-22 Melnyk V.I. (KWHA, s.n., 5 specimens) • Zakarpattia region, village Dilove, urochysche Kizi, lime slope: 1980-08-27 Tasenkevich L.O. (LWS 088718) • Zakarpattia region, Velikiy Bychkiv MK, urochysche Kuziy, lime rocks Sokolyne Berdo: 1991-08-11 Tasenkevich L.O. (LWS 088716) • Zakarpattia region, Tiachiv district, Uholka forestry, lime rock Hrebin, 820 m: 1988-07-28 Tasenkevich L.O. (LWS 088715) • Zakarpattia region, Tiachiv district, Carpathian Reserve, village Mala Uholka, lime rock Kopytsia: 1990-07-06 Tasenkevich L.O. (LWS 088714) • Zakarpattia region, Tiachiv district, Carpathian Reserve, lime rock Chury, village Mala Uholka: 1990-07-06 Tasenkevich L.O. (LWS 088712) • Zakarpattia region, Tiachiv district, Mala Uholka, Chur, Carpathian Reserve, lime slopes: 1990-07-06 Tasenkevich L.O. (LWS 088713) • Chernivtsi region, Putyla district, Chyvchyny, urochysche Chorniy Dil, Mt. Velikiy Kamin: 1978-07-14 Tasenkevich L.O. (LWS 007239, LWS 007240) • Zakarpattia region, Tiachiv district, Uholka forestry, lime rock Hrebin, 900 m a.s.l.: 1978-07-26 Tasenkevich L.O. (LWS 017268) • Zakarpattia region, Tiachiv district, Uholka forestry of Karpathian State Reserve, lime rock Velikiy Hrebin, 1050 m a.s.l., top, SW slope: 1979-08-13 Tasenkevich L.O. (LWS 017274) • Zakarpattia region, Tiachiv district, Uholka forestry of Karpathian State Reserve, lime rock Hrebin, 900 m a.s.l., SE slope: 1978-07-26 Tasenkevich L.O. (LWS 017268, LWS 017270, LWS 017271, LWS 017272, LWS 017275, LWS 017276) • Zakarpattia region, village Dilove, urochysche Kizi, lime rocks: 1980-08-27 Tasenkevich L.O. (LWS 017277, LWS 017278, LWS 017279, LWS 017280) • Zakarpattia region, Tiachiv district, Karpathian State Reserve, village Mala Uholka, lime rock Velikiy Hrebin: 1990-07-08 Tasenkevich L.O. (LWS 016632) • Chernivtsi region, Putyla district, Chyvchyny Mts., Mt. Velikiy Kamin, the summit: 1994-08-21 Chorney I.I. (CHER, s.n.) • Chernivtsi region, Putyla district, Chyvchyny Mts., Mt. Velikiy Kamin: 1991-07-18 Chorney I.I. (CHER, s.n.) • Chernivtsi region, Putyla district, Chyvchyny Mts., Chorniy Dil mountain range, Mt. Velikiy Kamin: 2003-09-21 Chorney I.I. et al. (CHER, s.n.) • Chernivtsi region, Putyla district, Chyvchyny Mts., Mt. Velikiy Kamin: 2004-08-10 Chorney I.I. et al. (CHER, s.n.).

##### Key to the species of *Galium mollugo* agg. in the Ukrainian Carpathians

**Table:** 

1	Stems few, ascendent or decumbent, glabrous or rarely slightly hairy at the base and/or in the nodes. Leaves oblanceolate to narrowly obovate, glabrous or rarely with sparse hairs underneath; cauline leaves up to 0.7 cm wide. Inflorescences lax	**2**
–	Stems usually numerous, erect or ascendent, glabrous or pubescent, usually becoming glabrescent in direction toward the apex. Leaves oblong to obovate, pubescent, rarely glabrous; cauline leaves up to 0.4–0.5 cm wide. Inflorescences dense	**3**
2	Stems normally 60–70 cm long, often much longer. Leaves 5–7 per whorl; basal leaves 1.2–3.0 (–3.5) cm long and 1.6–3.4 (–4.0) mm wide. Perianth (1.7–) 2.4–2.7 (–3.5) mm in diameter. Fruits (0.9–) 1.0–1.2 mm long, reddish-brown.	**G. mollugo subsp. mollugo (incl. *G. kernerianum* Klokov and *G. pseudomollugo* Klokov)**
–	Stems up to 40 (–45) long. Leaves 6–8 per whorl; basal leaves (0.5–) 1.0–1.2 cm long and 1.1–1.5 mm wide. Perianth 2.5–3.7 (–5.5) mm in diameter. Fruits 1.0–1.8 (–2.0) mm long, dark brown	**G. mollugo subsp. transcarpaticum**
3	Slender plants. Stem glabrous or sparsely hairy at the base and nodes. Stems 50–100 cm long, branched; lateral branches patent. Leaves oblong to oblanceolate, glabrous or rarely slightly hairy beneath. Pedicels patent or erect	**4**
–	Robust plants. Stem sparsely hairy at the base and nodes, rarely glabrescent. Stems 40–80 cm long, weakly branched; lateral branches extended and patent. Leaves narrowly obovate; usually pubescent on both surfaces, very rarely glabrous. Pedicels patent. Inflorescences with numerous flowers.	**G. album subsp. pycnotrichum (Heinr.Braun) Krendl**
4	Leaves oblong or oblanceolate, glabrous or rarely slightly hairy beneath; cauline leaves (1.4–) 1.7–2.0 (–2.5) cm long and 0.2–0.3 (–0.4) cm wide. Pedicels patent. Inflorescences with numerous flowers.	**G. album subsp. album**
–	Leaves narrowly oblanceolate, glabrous; cauline leaves (0.9–) l.2–1.5 (–1.8) cm long and 0.1–0.2 (–0.3) cm wide. Pedicels erect. Inflorescences with few flowers.	**G. album subsp. suberectum (Klokov) E. Michálk**.

## Conclusions

Due to the use of old herbarium material with degraded DNA, only a subset of the selected markers could be amplified successfully. In addition, the limited number of specimens analyzed restricts the strength of the taxonomic conclusions that can be drawn. A more robust assessment of *Galium transcarpaticum* will require broader population-level sampling based on freshly collected material and the application of a larger set of informative molecular markers or genome-wide data. Nevertheless, the molecular analyses conducted in the present study revealed a very close affinity between *G. transcarpaticum* and *G. mollugo*. The nucleotide dataset alone did not support the monophyly of *G. transcarpaticum*. Although several unique indels indicate some degree of molecular differentiation, the obtained evidence remains insufficient to confirm *G. transcarpaticum* as an independent species. Therefore, the taxon is more likely to represent a geographically and/or morphologically differentiated infraspecific lineage within the *G. mollugo* complex. We therefore reduce *G. transcarpaticum* to subspecific rank and propose the new combination and status G. mollugo subsp. transcarpaticum (Stojko & Tasenk.) A.V.Novikov, Nachychko & Zinenko, comb. et stat. nov. Considering the limitations listed above, the proposed subspecies rank is a conservative and integrative taxonomic solution, rather than a definitive resolution.

## Supplementary Material

XML Treatment for Galium mollugo subsp. transcarpaticum

## Additional information

### Artificial Intelligence (AI) use

The authors accept full responsibility for the content of the manuscript, including the disclosure of any use of AI.

No AI tools were used in the preparation of this manuscript.

### Author ORCIDs

A. Novikov https://orcid.org/0000-0002-0112-5070

V. Nachychko https://orcid.org/0000-0001-6756-2823

O. Zinenko https://orcid.org/0000-0001-5228-9940

### Data availability

All of the data that support the findings of this study are available in the main text or Supplementary Information.

## Supplementary materials

Supplementary material 1 Vouchers used for molecular study Data type xlsx https://binary.pensoft.net/file/1738163This dataset is made available under the Open Database License (http://opendatacommons.org/licenses/odbl/1.0/). The Open Database License (ODbL) is a license agreement intended to allow users to freely share, modify, and use this Dataset while maintaining this same freedom for others, provided that the original source and author(s) are credited. Andriy Novikov, Viktor Nachychko, Oleksandr Zinenko

Supplementary material 2 Primers and amplification used in molecular study Data type xlsx https://binary.pensoft.net/file/1738164This dataset is made available under the Open Database License (http://opendatacommons.org/licenses/odbl/1.0/). The Open Database License (ODbL) is a license agreement intended to allow users to freely share, modify, and use this Dataset while maintaining this same freedom for others, provided that the original source and author(s) are credited. Andriy Novikov, Viktor Nachychko, Oleksandr Zinenko

Supplementary material 3 Comparison of morphological characters of *G. transcarpaticum* and *G. mollugo* Data type xlsx https://binary.pensoft.net/file/1738165This dataset is made available under the Open Database License (http://opendatacommons.org/licenses/odbl/1.0/). The Open Database License (ODbL) is a license agreement intended to allow users to freely share, modify, and use this Dataset while maintaining this same freedom for others, provided that the original source and author(s) are credited. Andriy Novikov, Viktor Nachychko, Oleksandr Zinenko

Supplementary material 4 Data of *G. transcarpaticum* and *G. mollugo* specimens studied Data type xlsx https://binary.pensoft.net/file/1738166This dataset is made available under the Open Database License (http://opendatacommons.org/licenses/odbl/1.0/). The Open Database License (ODbL) is a license agreement intended to allow users to freely share, modify, and use this Dataset while maintaining this same freedom for others, provided that the original source and author(s) are credited. Andriy Novikov, Viktor Nachychko, Oleksandr Zinenko
